# Cwf16p Associating with the Nineteen Complex Ensures Ordered Exon Joining in Constitutive Pre-mRNA Splicing in Fission Yeast

**DOI:** 10.1371/journal.pone.0136336

**Published:** 2015-08-24

**Authors:** Noriko Sasaki-Haraguchi, Takeshi Ikuyama, Shogo Yoshii, Tomoko Takeuchi-Andoh, David Frendewey, Tokio Tani

**Affiliations:** 1 Department of Biological Sciences, Graduate School of Science and Technology, Kumamoto University, Kumamoto, Japan; 2 Regeneron Pharmaceuticals, Inc., Tarrytown, New York, United States of America; Keio University, JAPAN

## Abstract

Exons are ligated in an ordered manner without the skipping of exons in the constitutive splicing of pre-mRNAs with multiple introns. To identify factors ensuring ordered exon joining in constitutive pre-mRNA splicing, we previously screened for exon skipping mutants in *Schizosaccharomyces pombe* using a reporter plasmid, and characterized three exon skipping mutants named *ods1* (ordered splicing 1), *ods2*, and *ods3*, the responsible genes of which encode Prp2/U2AF^59^, U2AF^23^, and SF1, respectively. They form an SF1-U2AF^59^-U2AF^23^ complex involved in recognition of the branch and 3′ splice sites in pre-mRNA. In the present study, we identified a fourth *ods* mutant, *ods4*, which was isolated in an exon-skipping screen. The *ods4* ^+^ gene encodes Cwf16p, which interacts with the NineTeen Complex (NTC), a complex thought to be involved in the first catalytic step of the splicing reaction. We isolated two multi-copy suppressors for the *ods4-1* mutation, Srp2p, an SR protein essential for pre-mRNA splicing, and Tif213p, a translation initiation factor, in *S*. *pombe*. The overexpression of Srp2p suppressed the exon-skipping phenotype of all *ods* mutants, whereas Tif213p suppressed only *ods4-1*, which has a mutation in the translational start codon of the *cwf16* gene. We also showed that the decrease in the transcriptional elongation rate induced by drug treatment suppressed exon skipping in *ods4-1*. We propose that Cwf16p/NTC participates in the early recognition of the branch and 3′ splice sites and cooperates with the SF1-U2AF^59^-U2AF^23^ complex to maintain ordered exon joining.

## Introduction

In the processing of eukaryotic messenger RNA precursors (pre-mRNAs), removal of introns from pre-mRNAs (pre-mRNA splicing) is essential for gene expression. Pre-mRNA splicing takes place in a large ribonucleoprotein complex called a spliceosome, which consists of five small nuclear RNAs (snRNAs) named U1, U2, U4, U5, and U6 snRNA, and numerous protein factors (for a review, see [1,2]). The U1 small nuclear ribonucleoprotein (snRNP) binds to a 5′ splice site through the base pairing with the U1 snRNA. SF1 has been shown to bind to the branch site required for the formation of a lariat intermediate, while the U2AF large subunit (U2AF^65^ in mammals and U2AF^59^ in fission yeast) binds to the polypyrimidine tract [3]. In addition, the small subunit of U2AF (U2AF^35^ in mammals and U2AF^23^ in fission yeast) reportedly binds to AG at the 3′ splice site [4]. The early spliceosome assembly intermediate containing U1 snRNP and the U2AF^65^/U2AF^35^ heterodimer is called the E complex. U2 snRNP then joins the E complex to replace SF1 in an ATP-dependent manner, thereby forming a pre-spliceosome, the A complex [5]. The preassembled U4/U6·U5 tri-snRNP recognizes the A complex to form the precatalytic spliceosome, the B complex [6]. Subsequent activation of the spliceosome is associated with RNP rearrangements. The release of the U1 and U4 snRNPs from the B complex has been thought to be coincident with the joining of the Prp19-associated NineTeen Complex (NTC), which is required for the stable association of the U5 and U6 snRNPs to the complex, thereby activating the spliceosome [7]. The activated B* spliceosome then executes the first step of the splicing reaction, forming the C complex. After the second step of the reaction, the post-spliceosomal complex is generated to release the spliced mRNA [1].

In metazoans, splice site sequences are not sufficient for the recognition of exons and introns by the splicing machinery. Exonic or intronic *cis-*acting regulatory sequences and *trans*-acting factors regulate splice site selection. For example, SR proteins that contain one or two N-terminal RRMs (RNA-recognition motifs) and a C-terminal RS (arginine/serine-rich) domain were previously reported to bind to ESE (exonic splicing enhancer) sequences in order to regulate alternative and constitutive pre-mRNA splicing in coordination with other splicing factors [8].

Exons are ligated together through the splicing reaction, maintaining a reading frame to form a translatable mature mRNA. In humans, up to 95% of genes with multiple exons have been shown to undergo alternative pre-mRNA splicing to generate diverse isoforms from a single gene [9,10]. Specific sets of introns and exons are subjected to alternative pre-mRNA splicing under strict regulations [11]. In contrast, the majority of the remaining constitutively spliced exons in pre-mRNAs perform ordered 5′ to 3′ joining. It currently remains unclear how a cell ensures ordered exon joining in constitutive pre-mRNA splicing.

**Fig 1:**
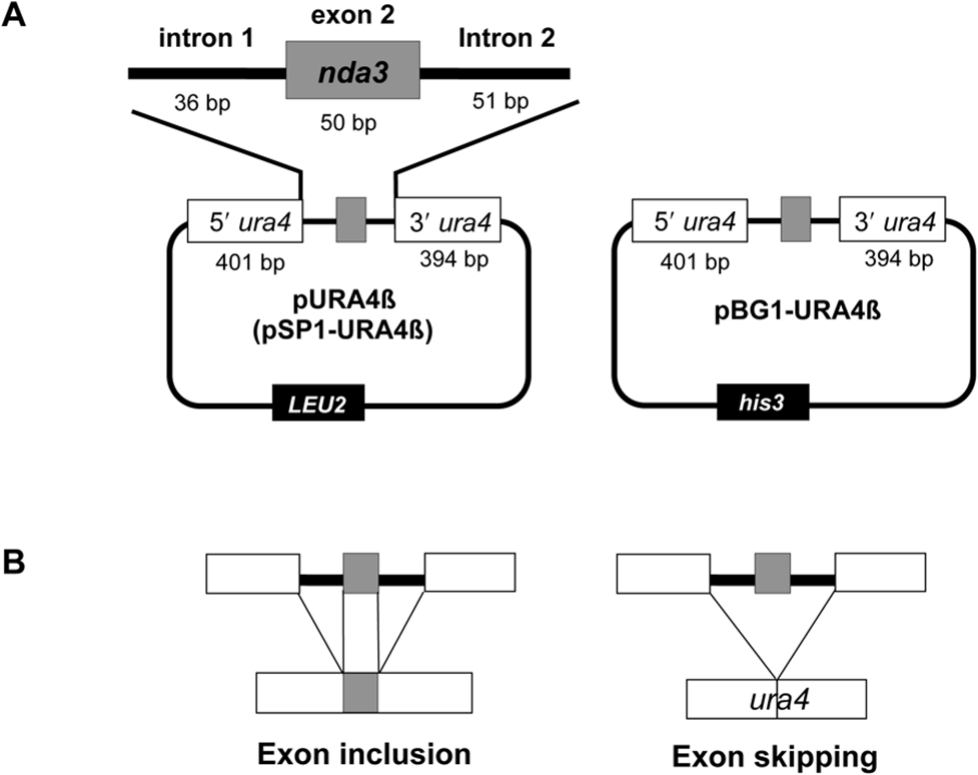
Structure of reporter plasmids for *ods* screening. (A) Structures of pURA4β (pSP1-URA4β) and pBG1-URA4β reporter plasmids. The pSP1 and pBG1 vectors have the *LEU2* and *his3* markers, respectively. The intron 1-exon 2-intron 2 region of the *S*. *pombe* β-tubulin gene (*nda3* ^*+*^) was amplified by PCR and inserted into the *Stu* I site in the *ura4* ^*+*^ gene. (B) Splicing patterns of the transcripts from the chimeric *ura4* gene in the reporter plasmids. Transcripts in which the internal exon is included produce non-functional Ura4p, whereas exon-skipped transcripts produce functional Ura4p.

We previously screened for mutants that caused exon skipping in fission yeast *Schizosaccharomyces pombe* using a pURA4β reporter plasmid to identify genes that maintain ordered exon joining in constitutive pre-mRNA splicing (Fig 1) [12]. Approximately 25% of genes have multiple introns in *S*. *pombe* [13,14], some of which were reported to undergo exon skipping type alternative splicing [15]. Therefore, *S*. *pombe* is a model organism suitable for analyzing the mechanisms responsible for maintaining ordered exon joining, compared to *Saccharomyces cerevisiae* that has a single intron in most intron-containing genes [16].

We previously identified three mutations that caused exon skipping: *ods1* (ordered splicing 1), *ods2*, and *ods3* [12]. The genes responsible for the mutations encode the subunits of the SF1-U2AF^59^-U2AF^23^ complex, suggesting that the initial recognition of the branch and 3′ splice sites is important for ensuring ordered exon joining in *S*. *pombe* [12]. In the present study, we characterized a fourth *ods* mutant, *ods4*, isolated by an exon-skipping screen. The *ods4* ^*+*^ gene was found to encode Cwf16p, a splicing factor associating with the NTC. Our results suggested that Cwf16p/NTC is involved in the initial co-transcriptional recognition of pre-mRNA to ensure ordered exon-joining in constitutive pre-mRNA splicing.

## Materials and Methods

### *S*. *pombe* Strains and General Methods

The *S*. *pombe* strains used in this study are listed in Table 1. The complete media YPD and YE [17,18] and minimum medium MM [17] were used for standard cultures of *S*. *pombe* strains. Appropriate growth supplements (uracil, leucine, and histidine) were added to MM. SPA medium was used for the induction of mating and sporulation of *S*. *pombe* [17]. The genetic methods used for *S*. *pombe* were as previously described [19].

**Table 1:** *S*. *pombe* strains used in this study.

Strain	Genotype	Reference/source
*UR470*	*h* ^*+*^, *leu1-32*, *ura4-D18*	S. Urushiyama
*UR471*	*h* ^*-*^, *leu1-32*, *ura4-D18*	S. Urushiyama
*UR502*	*h* ^*-*^, *leu1-32*, *ura4-D18*, *ade6-M216*	S. Urushiyama
*snh7*	*h* ^*-*^, *leu1-32*, *ura4-D18*, *ods4-1*	This study
*snh31*	*h* ^*-*^, *leu1-32*, *ura4-D18*, *ods1-3*	This study
*snh4*	*h* ^*-*^, *leu1-32*, *ura4-D18*, *ods1-1*	Haraguchi *et al*. 2007
*snh29*	*h* ^*-*^, *leu1-32*, *ura4-D18*, *ods1-2*	Haraguchi *et al*. 2007
*snh19*	*h* ^*-*^, *leu1-32*, *ura4-D18*, *ods1-2*	Haraguchi *et al*. 2007
*snh33*	*h* ^*-*^, *leu1-32*, *ura4-D18*, *ods1-2*	Haraguchi *et al*. 2007
*NH2*	*h* ^*+*^, *leu1-32*, *ura4-D18*, *his3-D1*	This study
*snh4-Mluh*	*h* ^*-*^, *leu1-32*, *ura4-D18*, *his3-D1*, *ods1-1*	This study
*snh29-Mluh*	*h* ^*-*^, *leu1-32*, *ura4-D18*, *his3-D1*, *ods1-2*	This study
*snh31-Mluh*	*h* ^*-*^, *leu1-32*, *ura4-D18*, *his3-D1*, *ods1-3*	This study
*snh19-Mluh*	*h* ^*-*^, *leu1-32*, *ura4-D18*, *his3-D1*, *ods2-1*	This study
*snh33-Mluh*	*h* ^*-*^, *leu1-32*, *ura4-D18*, *his3-D1*, *ods3-1*	This study
*snh7-Mluh*	*h* ^*-*^, *leu1-32*, *ura4-D18*, *his3-D1*, *ods4-1*	This study

### Plasmid Construction

The reporter plasmid pURA4β was previously constructed to screen for exon skipping mutants [12]. pSP1-URA4β was constructed from pURA4β by replacing a vector with the pSP1 vector [20] (Fig 1A). In the complementation cloning of the *ods4* ^*+*^ gene, we constructed pBG1-URA4β by replacing a vector portion of pSP1-URA4β with the pBG1 vector harboring the *his3* ^*+*^ marker [21]. To construct pMT-Cwf16-FLAG, the *cwf16* ^*+*^ gene that was C-terminally fused with a FLAG tag was cloned into the pMT plasmid containing the *ura4* ^*+*^ marker gene. pMT-mCwf16-FLAG containing the FLAG-tagged *cwf16* gene with the *ods4-1* mutation was generated using pMT-Cwf16-FLAG and a QuikChange II site-directed mutagenesis kit (Stratagene). The oligonucleotides used for mutagenesis are listed in Table 2.

**Table 2:** List of oligonucleotides.

Names	Sequences
***Primers for the RT-PCR analysis***	
oligo(dT)50	5'(50× t) 3'
tub-3	5' atatgcatctggtgtgtac 3'
tub-4	5' ctttggaagacatttcagc 3'
skipping-1	5' cgagggtattatacaaggcc 3'
act1-1	5’ tatgtgcaaagccggtttc 3’
act1-2	5’ tacctaccataataccatgg 3’
***Probe for exon-skipped products***	
ura4 probe	5' tctttgaggccttgtata 3'
***Primers for the construction of cwf16-FLAG***	
cwf16-1	5’ ctattcctgccattaatgcc 3’
cwf16-sma	5’ acccgggataccaaaaccttttc 3’
***Oligonucleotides for site directed mutagenesis***	
cwf16-mut1	5’ catagtttccaacattgtctgaacgaaag 3’
cwf16-mut2	5’ ctttcgttcagacaatgttggaaactatg 3’

### Backcrossing of *ura* ^+^ Mutants

Twenty-six *ura* ^*+*^ mutants harboring pURA4β were successively streaked on YPD plates several times to remove the pURA4β plasmid from cells. To determine the reproducibility of the *ura* ^*+*^ phenotype, each mutant was re-transformed with pURA4β and streaked on MM and MMU plates. The *ura* ^*+*^ mutants were crossed with a wild type strain (UR470 or UR471, Table 1) and tetrad analysis was performed. After re-transformation with pURA4β, three sets of tetrads were examined to see whether the *ura* ^*+*^ and *ura* ^*-*^ (wild-type) phenotypes segregated 2:2. Each mutant was backcrossed to wild type at least three times.

### Complementation Analysis

*snh7* and *snh31* harboring pSP1-URA4β were mated with *UR502* (a wild type strain), *ods1*, *ods2*, or *ods3* (Table 3) on a SPA plate. After 8 hours, cells were streaked on MMU plates to obtain diploid colonies. Each diploid was then streaked on MM and MMU plates and incubated at 26°C to examine phenotypic complementation.

**Table 3:** *S*. *pombe* strains used for the complementation analysis.

A	b
*snh7 (h* ^*+*^, *leu1-32*, *ura4-D18*, *ade6-M210*, *ods4-1*, *+pSP1-URA4β)*	*UR502 (h* ^*-*^, *leu1-32*, *ura4-D18*, *ade6-M216)*
	*ods1 (h* ^*-*^, *leu1-32*, *ura4-D18*, *ade6-M216*, *ods1-1)*
	*ods2 (h* ^*-*^, *leu1-32*, *ura4-D18*, *ade6-M216*, *ods2-1)*
	*ods3 (h* ^*-*^, *leu1-32*, *ura4-D18*, *ade6-M216*, *ods3-1)*
	*snh7 (h* ^*-*^, *leu1-32*, *ura4-D18*, *ade6-M216*, *ods4-1)*
*snh31 (h* ^*+*^, *leu1-32*, *ura4-D18*, *ade6-M210*, *ods1-3*, *+pSP1-URA4β)*	*UR502 (h-*, *leu1-32*, *ura4-D18*, *ade6-M216)*
	*ods1 (h* ^*-*^, *leu1-32*, *ura4-D18*, *ade6-M216*, *ods1-1)*
	*ods2 (h* ^*-*^, *leu1-32*, *ura4-D18*, *ade6-M216*, *ods2-1)*
	*ods3 (h* ^*-*^, *leu1-32*, *ura4-D18*, *ade6-M216*, *ods3-1)*
	*snh7 (h* ^*-*^, *leu1-32*, *ura4-D18*, *ade6-M216*, *ods4-1)*
	*snh31 (h* ^*-*^, *leu1-32*, *ura4-D18*, *ade6-M216*, *ods1-3)*

### Preparation of RNA and RT-PCR Analyses

Cells containing a reporter plasmid grown to a mid-log phase at an appropriate temperature were collected by centrifugation and washed twice with sterile water. Total RNAs were then prepared by the glass bead method [22]. After the treatment with RQ1 RNase free DNase (Promega) to remove contaminating genomic DNA, reverse transcription of isolated RNAs was performed using a PrimeScript RT-PCR Kit (TaKaRa Bio) with an oligo dT (50 mer) primer according to the manufacturer’s protocol. A PCR reaction was conducted in a 50 μl solution containing Ex Taq polymerase (2.5 units, TaKaRa Bio), 0.2 mM dNTP mix, 20 pmol forward primer, 20 pmol reverse primer, and 1 μl of reverse-transcribed cDNA. The primers used for RT-PCR are listed in Table 2. The tub-3 and tub-4 primers were complementary to the 3′ end of the *ura4* ^*+*^ first exon and 5′ end of the *ura4* ^+^ second exon, respectively. The skipping-1 primer was complementary to the sequence of the exon junction of the skipping spliced product. An RT-PCR amplification of *act1* mRNA was performed as a control. Amplified products were separated on a 5% polyacrylamide gel and stained with ethidium bromide (1μg/ml).

### Western Blot Analysis

In order to detect FLAG tagged Cwf16p, cells were cultured in MM + histidine for 24 hr at 30°C, pelleted, and resuspended in protein extraction buffer (25 mM Hepes, 120 mM NaCl, 5 mM MgCl_2_, 5% glycerol and 1 mM DTT). After vigorous mixing with glass beads at 4°C, samples were centrifuged twice at 9,100 g for 1 min at 4°C to prepare cell lysates. Protein lysates (250 ng/lane) were then electrophoresed on a 12% SDS–polyacrylamide gel and blotted onto a nylon membrane. The amounts of loaded proteins were validated by staining of gels with Coomassie Brilliant Blue. The blots were treated with an anti-FLAG M2 monoclonal antibody (Sigma-Aldrich), followed by a treatment with an anti-mouse IgG secondary antibody conjugated with HRP. Antibody binding was visualized with ECL Plus (GE Healthcare).

## Results

### Isolation of Novel *ods* Mutants

**Fig 2:**
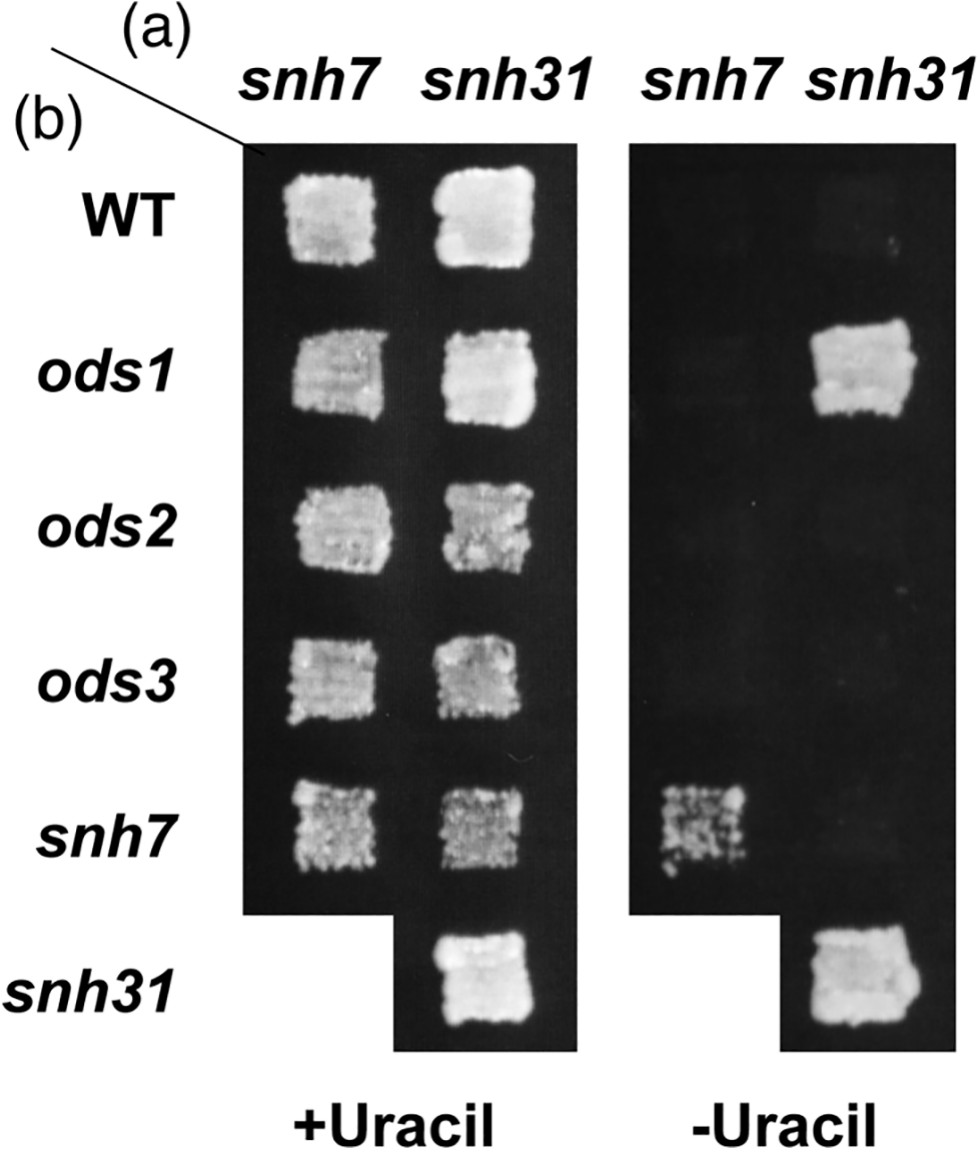
Complementation analysis of *snh7* and *snh31*. The isolated *ura* ^*+*^ mutants, *snh7* and *snh31*, were crossed with the wild-type haploid strain (*UR502*) or each of the isolated *ods* mutants to produce diploid strains. The resultant diploid strains were streaked on MMU (+Uracil) and MM (-Uracil) plates and then incubated at 26°C.

In a previous study [12], we isolated 34 *ura* ^*+*^ mutants that caused exon skipping in the splicing of URA4β pre-mRNA, which is transcribed from the reporter plasmid (pURA4β) (Fig 1). pURA4β contains a chimeric *ura4* ^+^ gene in which the intron 1-exon 2-intron 2 fragment of the *nda3* ^*+*^ gene was inserted in the middle of the *ura4* ^+^ gene. In screening, mutants that induced the exon skipping of reporter pre-mRNA produced a functional Ura4 protein and grew on a minimal medium (the *ura* ^+^ plate assay). Among the *ura* ^+^ mutants isolated, we analyzed eight that showed temperature-sensitive growth and identified the *ods1*, *ods2* and *ods3* genes, which encode the splicing factors U2AF^59^, U2AF^23^, and SF1, respectively [12]. To isolate a novel *ods* mutant, we analyzed the remaining 26 *ura* ^*+*^ mutants, tentatively named *snh*, that did not show a temperature-sensitive phenotype. Each mutant was backcrossed three times with a wild-type strain to remove extra mutations. Tetrad analyses of *snh7* and *snh31* showed 2:2 segregations of the *ura* ^+^ and *ura* ^*-*^ (wild-type) phenotypes, indicating that the *ura* ^*+*^ phenotype (exon-skipping phenotype) of these mutants was due to a single mutation (data not shown). The mutations in these mutants were recessive, as the heterozygous diploids with the wild-type alleles were unable to grow on the minimum plates (Fig 2, *snh7*/WT and *snh31*/WT).

We then performed complementation analyses with the previously identified exon-skipping mutants, *ods1*, *ods2*, and *ods3* to determine whether the isolated mutants contained novel exon-skipping mutations (Fig 2). The heterozygous diploids of *snh31* and *ods1* containing pSP1-URA4β exhibited the *ura* ^*+*^ phenotype, indicating that *snh31* belongs to the same complementation group as *ods1*. On the other hand, the heterozygous diploid of *snh7* and *ods1*, *ods2*, *ods3*, or *snh31* did not grow in the absence of uracil, suggesting that *snh7* belongs to a new complementation group. Therefore, we named *snh7* as the novel *ods* complementation group *ods4*. *ods4-1* grew very slowly at 22°C. This cold-sensitive phenotype of *ods4-1* appeared to be linked with the *ods* phenotype, as *ods4-1*, after backcrossing three times, still showed cold-sensitive growth.

As mentioned above, *snh31* was found to be allelic with *ods1*. The *ods1* gene was shown to encode the splicing factor Prp2p (U2AF^56^) [12]. A sequence analysis of the *prp2* gene in the *snh31* mutant revealed that the mutation site in this mutant is located in the start codon of the *prp2* gene, changing the start codon ATG to ATA. We named this allele *ods1-3*, *as* it was different from those of the previously identified *ods1-1* and *ods1-2* mutant alleles.

### Cloning of the *ods4* ^+^ Gene

**Fig 3:**
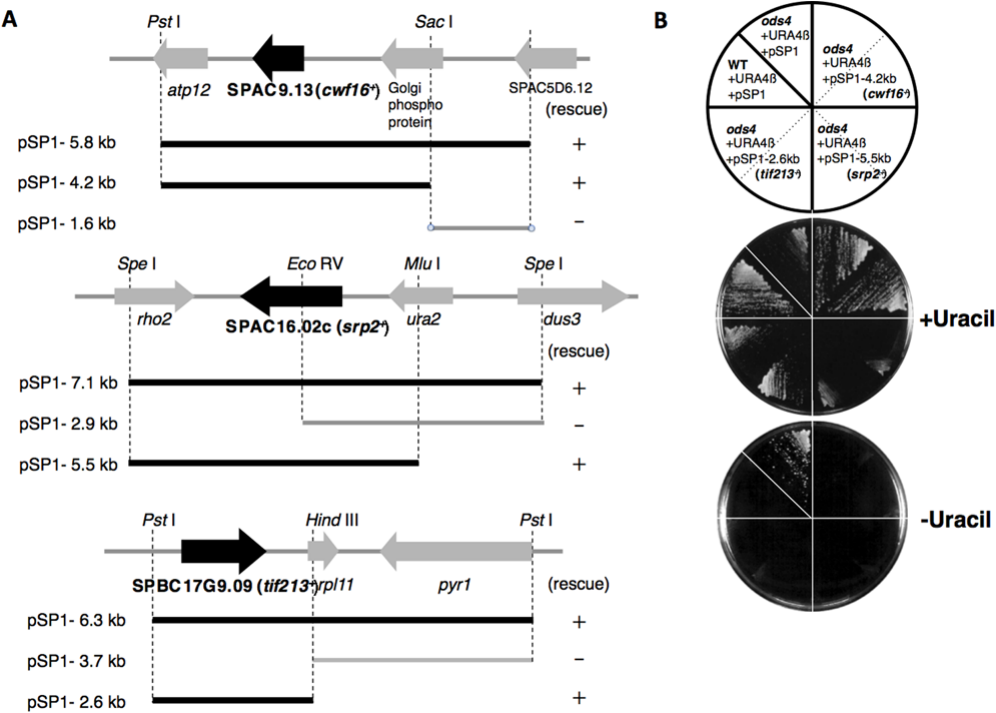
Cloning of the *ods4* ^+^ gene. (A) Restriction maps of the genomic DNA inserts in three plasmids complementing the *ods4-1* mutation. Schematic representations of subcloned fragments to identify the responsible gene are also shown. “+” shows that the fragments complemented the exon-skipping phenotype. (B) The DNA fragment containing the *cwf16* ^*+*^, *srp2* ^*+*^, or *tif213* ^*+*^ gene suppressed the exon-skipping (*ura* ^+^) phenotype of *ods4-1*. The transformants harboring a plasmid with each DNA fragment were streaked on MMU (+Uracil) or MM (-Uracil) plates and incubated at 26°C to examine the exon-skipping phenotype. Only *ods4-1* cells with pBG1-URA4β and the empty pSP1 vector survived on MM plates due to exon skipping of the reporter transcripts.

To clone the *ods4* ^*+*^ gene, we transformed the *ods4-1* mutant *(h* ^*-*^, *leu1-32*, *ura4-D18*, *ods4-1*) with an *S*. *pombe* genomic library constructed in pSP1. Screening for rescue of the *ods4-1* cold sensitive phenotype, we isolated three transformants that grew faster at 22°C than a control transformed with the pSP1 empty vector. The cold sensitive rescue transformants exhibited a *ura* ^+^ phenotype, suggesting that exon skipping of URA4β pre-mRNA occurred in the transformants. We then recovered plasmids from three transformants and subjected them to sequence analysis. The results obtained showed that the inserts in the plasmids were derived from the same genomic region. After the cloning of several DNA fragments, we identified a 4.2 kb *Pst* I-*Sac* I fragment that suppressed the *ura* ^*+*^ phenotype of *ods4-1* (Fig 3A). The fragment was found to contain an ORF encoding Cwf16p (complexed with Cdc5p) [23] associating with the NineTeen Complex (NTC) required for spliceosome activation [7, 24]. A sequence analysis of the *cwf16* gene in *ods4-1* revealed a single nucleotide change (A to T) in a start codon, indicating that a mutation in the *cwf16* ^+^ gene is responsible for the *ods4* mutant phenotype. Cwf16p was first reported to be a component of a multiprotein 40S complex containing Cdc5p, which is essential for pre-mRNA splicing [23], and later to be one of the NTC-associating proteins [25, 26].

In parallel with the cloning of the *ods4* ^+^ gene by complementation of the *cs* ^-^ phenotype, we also performed gene cloning using the exon-skipping (*ura* ^*+*^) phenotype as a complementation marker. We transformed the *ods4-1* mutant *(h* ^*-*^, *leu1-32*, *ura4-D18*, *his3-D1*, *ods4-1*) containing pBG1-URA4β, a reporter plasmid with the *his3* marker (Fig 1A). The *ods4-1* with pBG1-URA4β was transformed with an *S*. *pombe* genomic library constructed in the cosmid pSS10 [27]. The transformants (2,320 clones) were streaked on MM and MMU plates to identify the clones that rescued the exon-skipping phenotype, namely, clones that were unable to grow on MM plates. Through this negative screening, we isolated 50 *ura* ^*-*^ transformants that suppressed the *ods* phenotype. Cosmids were then recovered from *ura* ^*-*^ transformants and re-introduced into *ods4-1* to confirm that the *ura* ^*+*^ phenotype was actually suppressed.

Among the isolated cosmids, we selected cosmid-6 and cosmid-36 for further analyses because they showed different cutting patterns by restriction enzymes, suggesting that they contained DNA fragments from different genomic regions (data not shown). A subcloning analysis of cosmid-6 revealed that a 5.5 kb *Spe* I–*Mlu* I fragment contained a gene that suppressed the exon-skipping (*ura* ^*+*^) phenotype of *ods4-1* (Fig 3). The fragment included an ORF encoding Srp2p, one of the SR proteins involved in pre-mRNA splicing in *S*. *pombe* [28]. A sequence analysis showed that there were no mutations of the *srp2* ^*+*^ gene in *ods4-1*, indicating that Srp2p functions as a multi-copy suppressor for *ods4-1*.

On the other hand, a subcloning analysis of cosmid-36 revealed that a 2.6 kb *Pst* I-*Hin*d III fragment contained a gene that suppressed the exon-skipping (*ura* ^*+*^) phenotype of *ods4-1* (Fig 3). There is a single complete ORF encoding Tif213p in that subcloned fragment. A sequence analysis of the *tif213* ORF and the 3′ and 5′ UTRs (500 bp) in *ods4-1* showed no mutations, indicating that Tif213p was also a multi-copy suppressor for *ods4-1*. Tif213p is the gamma subunit of the translation initiation complex eIF2 (eIF2γ) that is considered to play a role in recruitment of tRNA-Met to the start codon in *S*. *pombe* [29]. The involvement of Tif213p in the pre-mRNA splicing reaction has not yet been reported.

**Fig 4:**
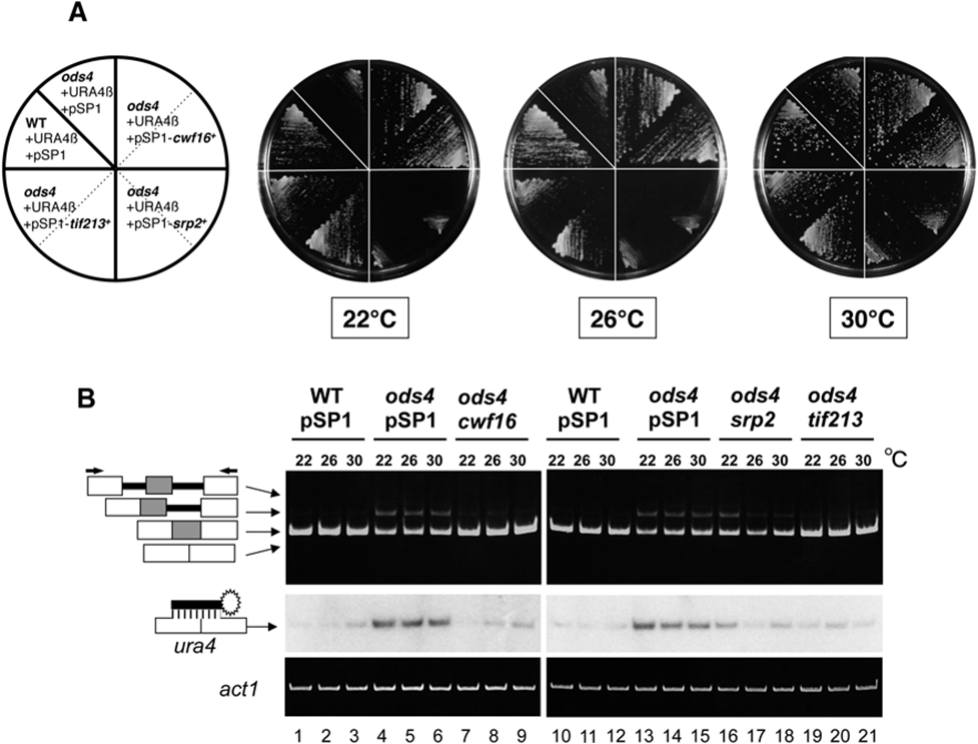
Overexpression of the *cwf16* ^*+*^, *srp2* ^*+*^, or *tif213* ^*+*^ gene suppressed exon skipping in *ods4*-*1*. (A) The overexpression of the *cwf16* ^+^ or *tif213* ^+^ gene rescued the *cs* phenotype of *ods4-1*. Transformants with pBG1-URA4β and the *cwf16* ^*+*^, *srp2* ^*+*^, or *tif213* ^*+*^ plasmid were streaked on MMU plates and incubated at 22, 26, or 30°C to test their complementarity for the *cs* phenotype of *ods4-1*. Transformants with the *cwf16* ^*+*^ or *tif213* ^*+*^ plasmid grew well at 22°C, whereas *ods4-1* itself showed slow growth at the same temperature (*ods4*+URA4β+pSP1). The overexpression of Srp2p resulted in slow growth at all temperatures. (B) Total RNAs were isolated from wild type (WT), *ods4-1*, and *ods4-1* with the *cwf16* ^*+*^, *srp2* ^*+*^, or *tif213* ^+^ plasmid, and subjected to RT-PCR analyses. All transformants contained pBG1-URA4β in addition to the rescued genes or pSP1 vector as indicated. Amplified cDNA products were electrophoresed on a 5% acrylamide gel, stained with ethidium bromide (upper panel), and then subjected to a Southern blot analysis using an oligonucleotide probe that specifically hybridizes to the exon-skipped product (middle panel). RT-PCR of *act1* ^*+*^ mRNA was performed as a control (lower panel). The structures of the RT-PCR products confirmed by the sequence analysis are shown on the left. Arrows indicate the positions of the tub-3 and tub-4 primers used for RT-PCR analyses.

To determine if the overexpression of Srp2p and Tif213p also suppressed the *cs* ^*-*^ phenotype of *ods4-1*, cells harboring pBG1-URA4β and a multicopy plasmid expressing Srp2p or Tif213p were streaked on plates containing uracil, and incubated at 22, 26, and 30°C (Fig 4A). The growth of *ods4-1* at 22°C was slower than that of the wild-type cells (the *cs* ^-^ phenotype). When Tif213p was overexpressed in *ods4-1*, cells grew well at 22°C, similar to the overexpression of Cwf16p. This result indicated that the overexpression of Tif213p suppressed the *cwf16* mutation. On the other hand, the overexpression of Srp2p did not complement the *cs* ^*-*^ phenotype of *ods4-1*. Even at a permissive temperature of 30°C, transformants with pSP1-*srp2* ^*+*^ showed impaired growth (Fig 4A), suggesting that the overexpression of Srp2p was toxic to *S*. *pombe* cells.

To confirm that the *ura* ^*+*^ phenotype was induced by the suppression of the exon skipping, we performed RT-PCR and Southern blot analyses to detect exon-skipping products in *ods4-1* cells in which the *cwf16* ^*+*^, *srp2* ^*+*^, or *tif213* ^*+*^ gene was overexpressed from the plasmid (Fig 4B). In *ods4-1* cells, the exon-skipping product was detected at all temperatures, whereas the amount of the skipping product was very low in the wild-type cells (Fig 4B, middle panel, lanes 1–6 and 10–15), indicating a direct correlation of the *ura* ^*+*^ phenotype with the exon skipping of URA4β pre-mRNA. In addition, *ods4-1* accumulated a partially spliced product containing the *nda3* ^*+*^ intron 2 (Fig 4B, upper panel, lanes 4–6 and 13–15), suggesting that *ods4-1* has a weak splicing defect. Southern blot analyses also showed that the overexpression of Cwf16p, Srp2p, or Tif213p suppressed the exon skipping of URA4β pre-mRNA in *ods4-1* (Fig 4, middle panel, lanes 7–9 and 16–21).

### Srp2p Is a Multi-Copy Suppressor for *ods* Mutants

**Fig 5:**
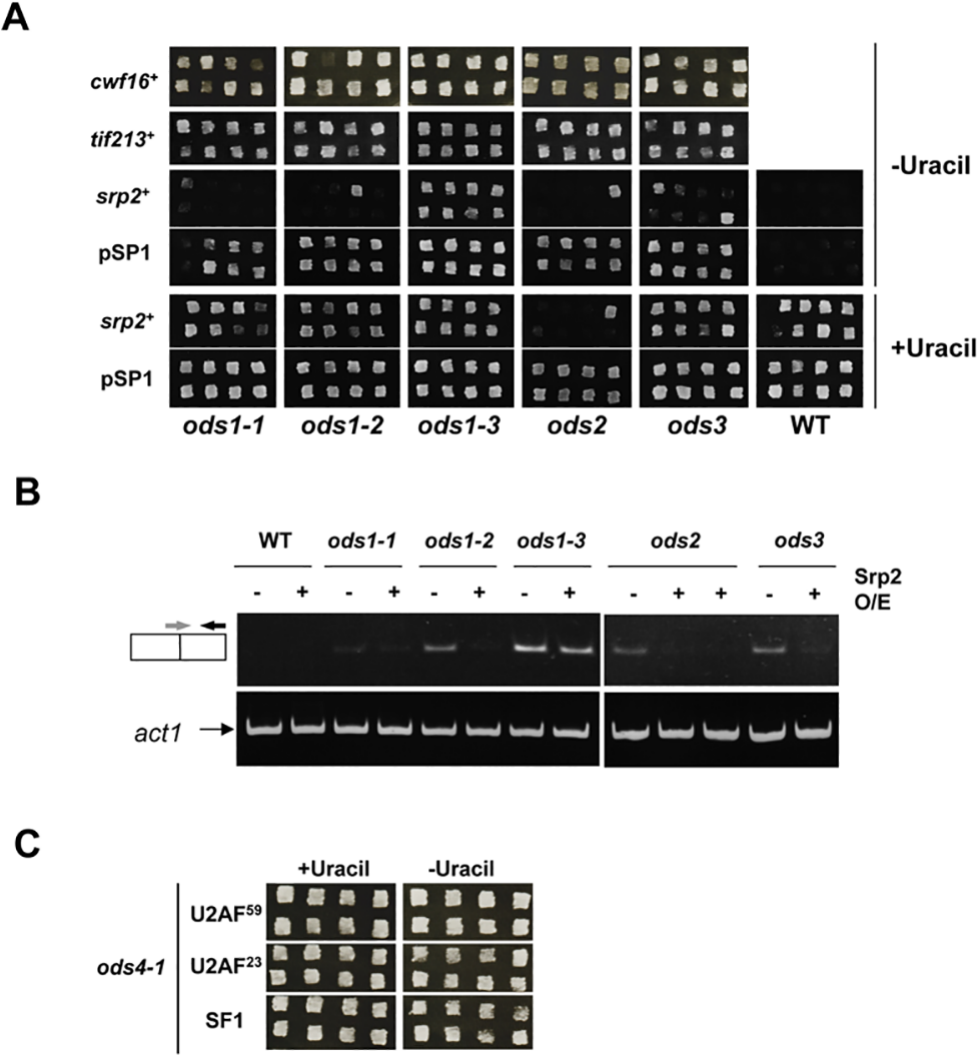
Functional complementarities of *ods* and suppressor genes. (A) The WT strain and *ods* mutants were transformed with pBG1-URA4β and pSP1*-srp2* ^*+*^ or pSP1. Eight independent transformants were patched on MMU (+Uracil) and MM (-Uracil) plates and incubated at 26°C. Cwf16^+^ and tif213^+^ transformants grew well on MM (-Uracil) plates, suggesting that the overexpression of Cwf16p and Tif213p did not complement the exon-skipping (*ura* ^+^) phenotype of previously isolated *ods* mutants. In contrast, most transformants with pSP1*-srp2* ^*+*^, except for *ods1-3* transformants, were unable to grow on MM plates, suggesting that the overexpression of Srp2p suppressed the exon skipping of URA4β pre-mRNA. Several *ods* transformants grown on MM plates may reflect the low copy numbers of pSP1*-srp2* ^*+*^ in those clones. pSP-*srp2* ^*+*^ could not suppress the *ods1-3* mutation, which has the ATA-mutated start codon. (B) The RT-PCR analysis to detect exon-skipping products in the transformants. RT-PCR was carried out using a skipping-1 primer with a sequence complementary to the spliced product and spanning the spliced junction. RT-PCR of *Act1* mRNA was also performed as a control. (C) *ods4-1* was transformed with pBG1-URA4β and the plasmid containing the *ods1* ^+^ (U2AF^59^), *ods2* ^+^ (U2AF^23^), or *ods3* ^+^ (SF1) gene. The transformants grew well on MM (-Uracil) plates, suggesting that U2AF^59^, U2AF^23^, and SF1 had no suppressor activity against *ods4-1*.

In order to examine if the overexpression of Cwf16p, Srp2p, or Tif213p suppressed not only the *ods4-1* mutation, but also the other previously identified exon-skipping mutations, *ods1*, *ods2*, and *ods3* [12], we introduced pBG1-URA4β and the plasmid carrying each gene into the corresponding mutant and then observed their growth on MMU (+Uracil) or MM (-Uracil) plates. As shown in Fig 5A, the *ods1*, *ods2*, and *ods3* mutants with the plasmid expressing Cwf16p and Tif213p grew well on MM (-Uracil) plates, suggesting that the overexpression of these two proteins did not suppress the exon-skipping (*ura* ^+^) phenotype of the other *ods* mutations. In contrast, *ods1-1*, *ods1-2*, *ods2-1*, *and ods3-1* cells overexpressing Srp2p did not grow on MM (-Uracil) plates, suggesting that exon skipping was suppressed by the overexpression of Srp2p in these mutants. Although a few *srp2* ^+^ transformants grew on MM plates, they may have grown due to the low copy number of the introduced pSP1-*srp2* ^+^ in these clones. Srp2p did not suppress the *ods1-3* mutation, showing its allele-specific suppression in the case of *ods1*. RT-PCR analyses of the transformants (Fig 5B) showed that overexpression of Srp2p repressed the exon skipping of URA4β pre-mRNA in *ods1-1*, *ods1-2*, *ods2*, and *ods3* but not in *ods1-3*, consistent with the growth assays (Fig 5A).

We also tested if the *ods4-1* mutation was rescued by the overexpression of products from other *ods* ^*+*^ genes. We transformed *ods4-1* with pBG1-URA4β and plasmids carrying the *ods1* ^+^ (U2AF^59^), *ods2* ^+^ (U2AF^23^), or *ods3* ^+^ (SF1) genes and subjected the transformants to the *ura* ^+^ plate assay. The results obtained showed that all transformants grew well on MM plates, suggesting that none of the other *ods* ^+^ genes can serve as a multicopy suppressor for *ods4-1* (Fig 5C).

### Overexpression of Tif213p Promoted Translational Initiation of Mutated *cwf16* mRNA

**Fig 6:**
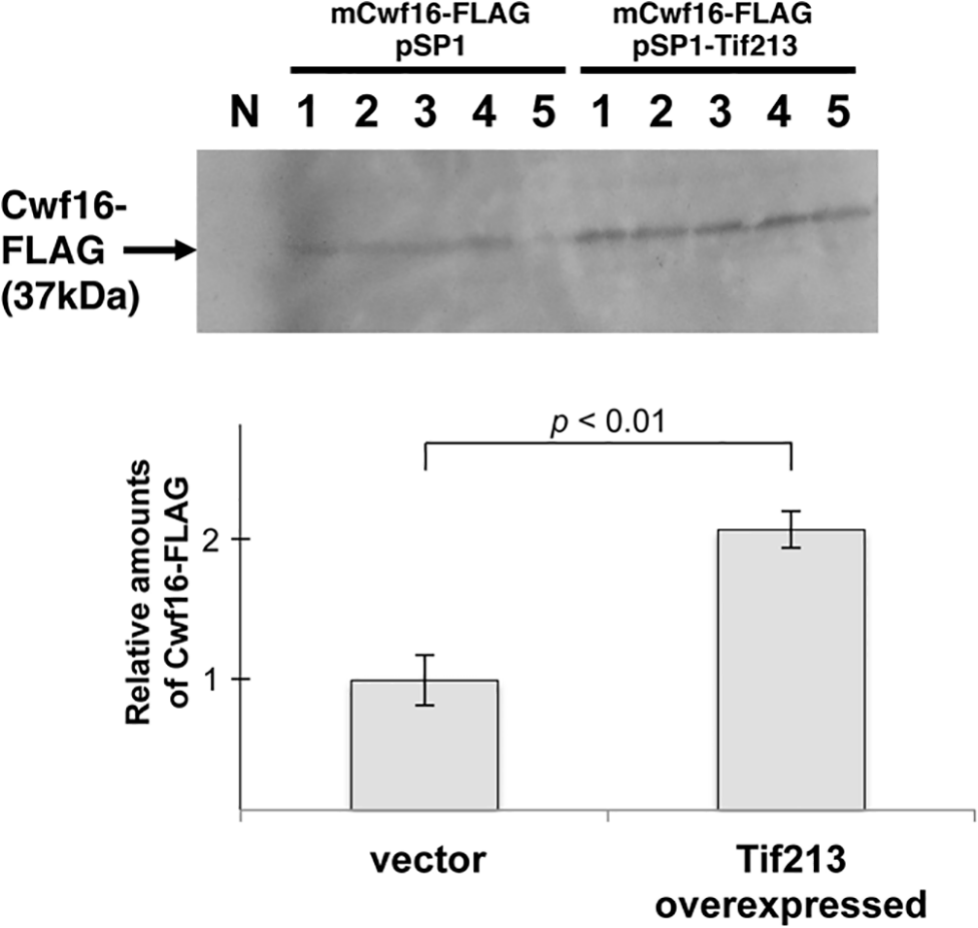
Overexpression of Tif213p promoted translation of *cwf16* mRNA containing a mutated start codon in *ods4-1*. *ods4-1* was transformed with pMT-mCwf16-FLAG containing the mutated start codon (the *ods4-1* mutation). pSP1-Tif213 or pSP1 was simultaneously transformed. Five independent transformants were cultured and subjected to a western blot analysis using an anti-FLAG antibody. The intensity of each band was quantitated with a FUJIFILM LAS-1000 and graphed (lower panel).

In *Saccharomyces cerevisiae*, an eIF2γ mutant *sui4* has been shown to initiate the translation of *HIS4* mRNA from the unusual mutated start codon UUG [30]. The *ods4-1* mutant had the same mutation in the start codon of the *cwf16* ^+^ gene, changing ATG to TTG. We identified Tif213p, an *S*. *pombe* homologue for the eIF2γ subunit, as a multi-copy suppressor of *ods4-1*. To examine if overexpression of Tif213p promotes the translational initiation of the mutated *cwf16* mRNA, we performed a western blot analysis for Cwf16p using plasmids expressing FLAG-tagged Cwf16p mRNA with the A to U mutation in the start codon (mCwf16). *ods4-1* cells expressing FLAG-tagged Cwf16p grew well at 22°C, indicating that FLAG-tagged Cwf16p is functional and complemented the *cs* phenotype of *ods4-1* (data not shown). As shown in Fig 6, western blot analysis revealed that overexpression of Tif213p increased the amount of Cwf16p-FLAG translated from the mutated mRNA. The size of Cwf16p-FLAG translated from the mRNA with the mutant start codon (37 kDa) was the same as that from the wild-type Cwf16p-FLAG mRNA, suggesting that the UUG codon in *mCwf16* mRNA was used as a start codon to translate Cwf16-FLAG. These results suggested that Tif213p suppressed the *ods4-1* mutation by enhancing translational initiation rather than by activating splicing.

### Decrease in the Elongation Rate in Transcription Induced Repression of Exon Skipping

**Fig 7:**
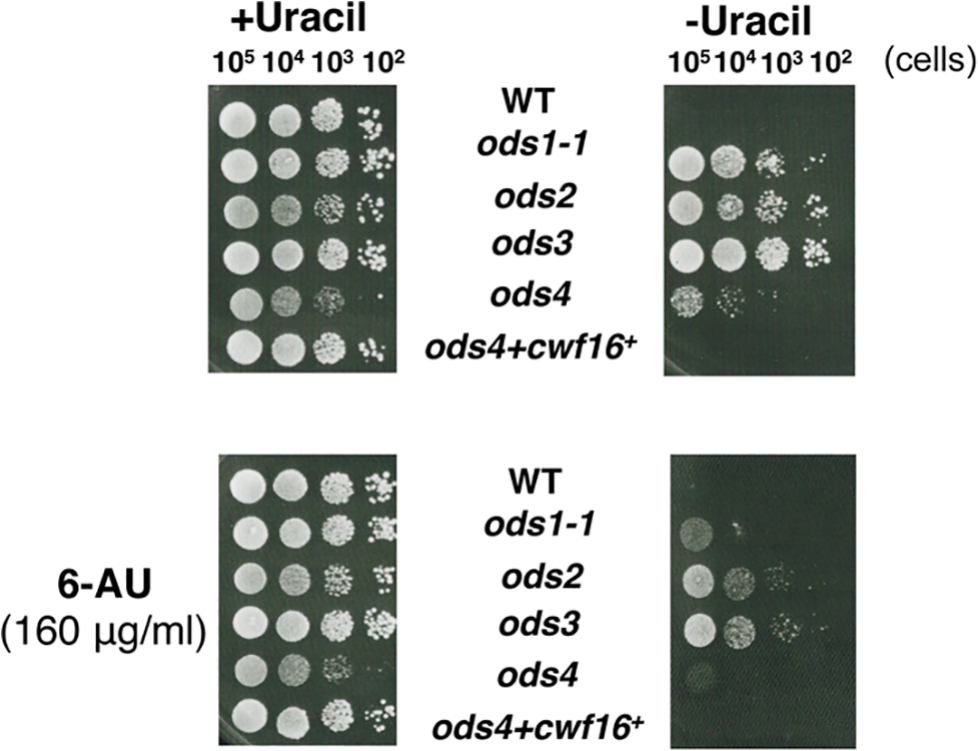
Treatment of *ods4-1* cells with 6-AU suppressed the exon-skipping phenotype. Serially diluted *ods4-1* and wild-type cells harboring pURA4β were spotted on MMU (+Uracil) and MM (-Uracil) plates with 160 μg/ml of 6-AU (+6-AU, lower panel) or without 6-AU (upper panel), and incubated at 26°C for 5 days. The growth of *ods4-1* and other *ods* mutants on MM (-Uracil) was impaired in the presence of 6-AU, whereas their growth on MMU (+Uracil) was not affected under the same conditions, suggesting that the exon skipping of URA4β pre-mRNA was suppressed by the treatment with 6-AU.

A previous study reported that the splicing reaction is coupled with transcription, and the transcription rate of RNA polymerase II affects alternative pre-mRNA splicing [31]. In *S*. *cerevisiae*, the treatment of cells with 6-azauracil (6-AU) or mycophenolic acid (MPA), which reduce the transcription elongation rate, was found to repress exon skipping [32]. 6-AU and MPA inhibit nucleotide biosynthesis, leading to the depletion of the cellular nucleotide pools necessary for efficient transcription by RNA polymerase [32]. We previously reported that a treatment with 6-AU or MPA reduced the exon skipping of URA4β pre-mRNA in *ods1*, *ods2*, and *ods3* mutants, suggesting that the relative rates of transcription elongation and splicing affected the fidelity of ordered exon joining in the *ods* mutants [12]. To test for the same effect in *ods4-1*, we spotted *ods4-1* cells with pURA4β on plates containing 6-AU and incubated them at 26°C. If the drug repressed exon skipping, the cells would be unable to grow on MM (–Uracil) plates. As shown in Fig 7, 6-AU severely reduced the ability of *ods4-1* containing pURA4β to grow in the absence of uracil, suggesting that the exon skipping of URA4β pre-mRNA was suppressed by a reduced rate of transcriptional elongation.

## Discussion

To understand the molecular mechanism that ligates successive exons orderly in constitutive pre-mRNA splicing, we previously screened for exon-skipping mutants in *S*. *pombe* and identified three temperature-sensitive mutations (*ods1*, *ods2*, and *ods3*) that caused exon skipping in *S*. *pombe* [12]. In the present study, we characterized a fourth *ods* mutant, *ods4*, that caused exon skipping of transcripts from the reporter plasmid.

We found that the *ods4* ^*+*^ gene encodes Cwf16p, which associates with the NTC (Prp19-associated NineTeen Complex). In *S*. *cerevisiae*, the NTC consists of eight core proteins (PRP19, CEF1/CDC5, CLF1, SYF1, SYF2, ISY1, NTC25, and NTC20) and more than a dozen NTC-associating proteins including Yju2p, an *S*. *cerevisiae* homologue of Cwf16p [24]. The NTC-associating proteins show dynamic associations with the NTC and spliceosome [24]. The NTC has been thought to bind to the spliceosome after the release of the U1 and U4 snRNPs to stabilize the association of the U5 and U6 snRNPs with the spliceosome [7]. However, a recent analysis of the purified A complex (pre-spliceosome) assembled during the early stage of the splicing reaction showed that it already contained the NTC proteins in addition to U1 and U2 proteins in the absence of U4/U6 snRNPs [33]. In addition, the NTC was shown to interact with U2AF^65^ (the large subunit of U2AF), which binds directly to the phosphorylated C-terminal domain (CTD) of RNA polymerase II [34]. The interaction between the NTC and RNA polymerase II promoted the recruitment of U2AF^65^ and the NTC to nascent pre-mRNAs, leading to transcription-coupled pre-mRNA splicing. These reports support the idea that Cwf16p/NTC is involved in the early step of the splicing reaction.

**Fig 8:**
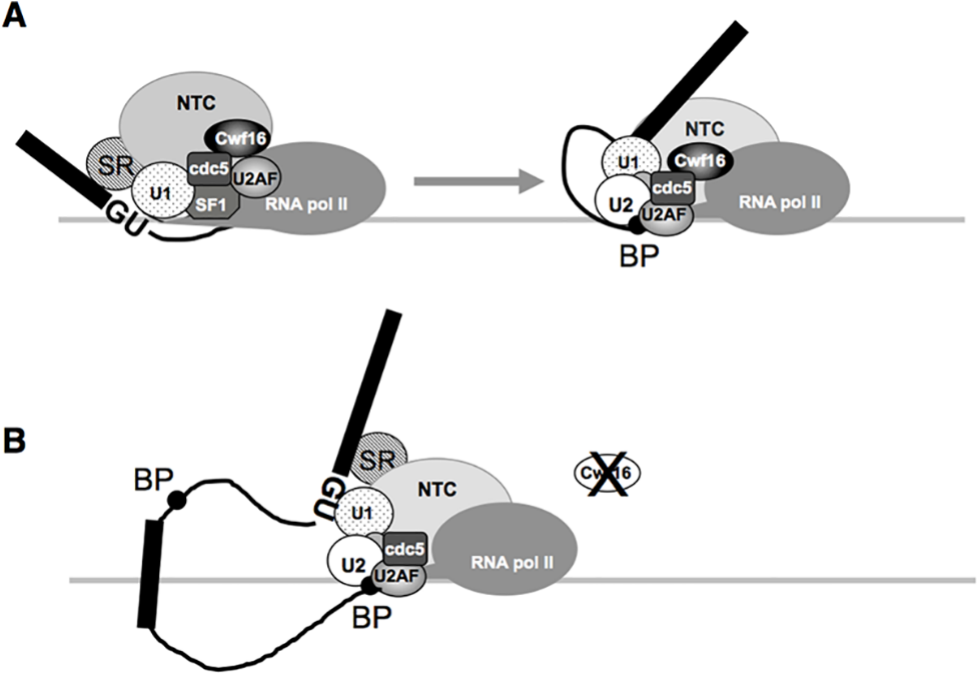
A transcription-coupled ordered splicing model. (A) The 5′ splice site in the nascent transcript is recognized by U1 snRNP associating with the CTD of elongating RNA polymerase II and the SF1-U2AF^59^-U2AF^23^/NTC- Cwf16 complex. After transcription proceeds, a branch point sequence (BP) is recognized by the U1/SF1-U2AF^59^-U2AF^23^/NTC-Cwf16 complex to form a pre-spliceosome. (B) The depletion of Cwf16p by the *ods4-1* mutation induces the weakened recognition of the branch and 3′ splice sites by the U1/SF1-U2AF^59^-U2AF^23^/NTC complex and increases an opportunity to cause exon skipping, thereby ligating to the far downstream exon.

The treatment of cells with 6-AU, which slows down the elongation of transcription, suppressed exon skipping in *ods4-1* (Fig 7). This result suggested that the co-transcriptional recognition of a nascent pre-mRNA by the splicing machinery is important for maintaining ordered 5' to 3' exon joining, thereby providing further evidence to support the "first come, first served" model based on the co-transcriptional recognition of splice sites [35]. Previous studies suggested that U1 snRNP associates with the SF1-U2AF^59^-U2AF^23^ complex and NTC/Cwf16, and also binds to the phosphorylated CTD of RNA polymerase II [34,36,37]. Therefore, it is possible that the complex consisting of U1 snRNP, SF1-U2AF^59^-U2AF^23^, and NTC/Cwf16 recognizes a 5′ splice site in a nascent pre-mRNA through base pairing with U1 snRNA (Fig 8A). After the branch and 3′ splice sites appear in pre-mRNA as transcription proceeds, they are recognized by the U1/SF1-U2AF^59^-U2AF^23^/ NTC-Cwf16 complex to form a pre-spliceosome bridging the two ends of the intron; exons are then ligated orderly (Fig 8A). On the other hand, in the *ods4-1* mutant, the depletion of Cwf16p by the nonsense mutation may affect the fidelity of splice site recognition by the U1/SF1-U2AF^59^-U2AF^23^/NTC complex, causing skipping of the first available exon and ligation to the next exon in some pre-mRNA molecules (Fig 8B). Further analyses are now in progress to elucidate the roles of Cwf16p in recognition of the branch and 3′ splice sites in concert with the SF1-U2AF^59^-U2AF^23^ complex in the transcription-coupled splicing reaction.

During screening of the gene responsible for the *ods4-1* mutation, we identified Tif213p and Srp2p as multicopy suppressors of the *ods4-1* mutation. Tif213p is an *S*. *pombe* homologue of the γ subunit of the translation initiation factor eIF2 [38]. It was unexpected that the identified suppressor was an essential factor for translational initiation. In *S*. *cerevisiae*, the *sui4* suppressor mutation encoding a homologue of Tif213p was shown to induce initiation of translation using a mutated UUG codon instead of a canonical AUG start codon in the *HIS4* locus [30,39]. Interestingly, a mutation found in the *cwf16* gene in *ods4-1* changed the AUG start codon to UUG. A western blot analysis revealed that the translation of Cwf16 was enhanced by the overexpression of Tif213p (Fig 6). Therefore, excess Tif213p expressed from the multicopy plasmid may enhance the translational initiation of the A to U mutated Cwf16 mRNA to produce functional Cwf16p, thereby suppressing exon skipping in this mutant. This is compatible with Tif213p only suppressing the *ods4-1* mutation, which has the TTG mutation in the start codon, among the four *ods* mutations (Fig 5).

Srp2p, another multi-copy suppressor for *ods4-1*, is an *S*. *pombe* member of the family of SR proteins, which contain two RNA-binding domains at their N-termini followed by a short serine/arginine (SR) repeat [28]. In mammals, SR proteins are known to play major roles in the regulation of constitutive and alternative pre-mRNA splicing through their binding to exonic or intronic splicing enhancers (for a review, see [40]). Several lines of evidence in mammals indicated that the overexpression of SR proteins promoted proximal 5′ splice site selection in pre-mRNAs with multiple 5′ splice sites [41]. These properties are consistent with our observation that overexpression of Srp2p inhibited exon skipping and promoted orderly splicing (Fig 4B). Thus, the *S*. *pombe* Srp2p appears to function in pre-mRNA splicing in a manner similar to its mammalian orthologs.

We found that Srp2p functioned as a multicopy suppressor for all *ods* mutations, except *ods1-3* (Fig 5). *ods1-3* has a G to A mutation in the start codon of the U2AF^59^ gene, changing the ATG start codon to ATA. As U2AF^59^/Prp2p is essential for pre-mRNA splicing, the U2AF^59^ mRNA with the mutated AUA codon may be translated with low efficiency. It is likely that the overexpression of Srp2p cannot complement the low level of U2AF^59^. On the other hand, the other *ods1* allele, *ods2-1*, and *ods3-1* have a missense point mutation in each responsible gene, generating the mutated protein, and appear to be functionally rescued by the overexpression of Srp2p. We found that the overexpression of Srp2p caused severe growth impairments in *ods2*, the responsible gene of which encodes the small subunit of U2AF (U2AF^23^), suggesting that excess Srp2p was toxic for S. *pombe* cells in the presence of mutated U2AF^23^ (Fig 5A). This result is compatible with the fact that Srp2p interacts with U2AF^23^ in *S*. *pombe* [42]. Srp2p is also known to associate with Cdc5p, a core component of the NTC [43]. Thus, it is possible that excess Srp2p suppresses exon skipping through its interaction with a pre-mRNA and the U1/SF1-U2AF^59^-U2AF^23^/NTC complex in the *ods4-1* mutant.
